# Optical quantum nondemolition measurement of a single rare earth ion qubit

**DOI:** 10.1038/s41467-020-15138-7

**Published:** 2020-03-30

**Authors:** Mouktik Raha, Songtao Chen, Christopher M. Phenicie, Salim Ourari, Alan M. Dibos, Jeff D. Thompson

**Affiliations:** 1 Department of Electrical Engineering, Princeton University, Princeton, NJ 08544 USA; 2 Present Address: Nanoscience and Technology Division, Argonne National Laboratory, Argonne, IL 60439 USA

**Keywords:** Atomic and molecular interactions with photons, Photonic devices, Quantum optics, Qubits

## Abstract

Optically-interfaced spins in the solid state are a promising platform for quantum technologies. A crucial component of these systems is high-fidelity, projective measurement of the spin state. Here, we demonstrate single-shot spin readout of a single rare earth ion qubit, Er^3+^, which is attractive for its telecom-wavelength optical transition and compatibility with silicon nanophotonic circuits. In previous work with laser-cooled atoms and ions, and solid-state defects, spin readout is accomplished using fluorescence on an optical cycling transition; however, Er^3+^ and other rare earth ions generally lack strong cycling transitions. We demonstrate that modifying the electromagnetic environment around the ion can increase the strength and cyclicity of the optical transition by several orders of magnitude, enabling single-shot quantum nondemolition readout of the ion’s spin with 94.6% fidelity. We use this readout to probe coherent dynamics and relaxation of the spin.

## Abstract (web summary)

Individually addressed rare earth atoms in solid crystals are an emerging platform for quantum information processing. Here the authors demonstrate a key requirement, by realizing single-shot, quantum non-demolition measurements of the spin of single Er^3+^ ions in Y_2_SiO_5_ crystals with nearly 95% fidelity.

## Introduction

Atomic and atom-like defects in the solid state provide an optical interface to individual electronic and nuclear spin qubits^1^, and are used for a variety of quantum technologies. As sensors, they can probe temperature and magnetic and electric fields with nanoscale spatial resolution^2–4^. In quantum networks, spin-photon entanglement^5–7^ has enabled deterministic entanglement of remote spins^8^. Defect spins have also been used to demonstrate key components of quantum information processors, including quantum error correction^9^ and 10-qubit quantum registers with multi-qubit gates^10^.

These works primarily leverage the well-studied nitrogen vacancy (NV) center in diamond. However, a much broader range of defects exists that may be advantageous for particular applications. For example, the SiV^−^^11^ and SiV^0^ ^12^ color centers in diamond are promising for quantum networks because of their low spectral diffusion, while color centers in silicon carbide^13^ may be easier to integrate with nanoscale devices. Rare earth ions are another family of defects that can offer long spin coherence^14^ and narrow, stable optical transitions (in the telecom band for the case of Er^3+^)^15^, and may be doped into a variety of host crystals. Several recent works have begun to probe individual rare earth ions^16–21^, using an optical cavity to overcome their low intrinsic photon emission rates^20,21^.

A key capability for atomic defects is high-fidelity spin readout using the optical transition^1^. Single-shot optical spin measurements have been achieved in quantum dots^22^ and in the NV^23^ and SiV^−^^24^ color centers in diamond by leveraging highly cyclic optical transitions that arise from atomic selection rules. However, cyclic optical transitions are not a universal feature of atomic defects, and are often absent in low-symmetry defects and in the presence of strain^25^ or spin-orbit coupling without careful alignment of the magnetic field^22,24^. Single-shot readout has not been achieved in atomic defects without intrinsic cycling transitions, such as rare earth ions^26^.

**Fig. 1:**
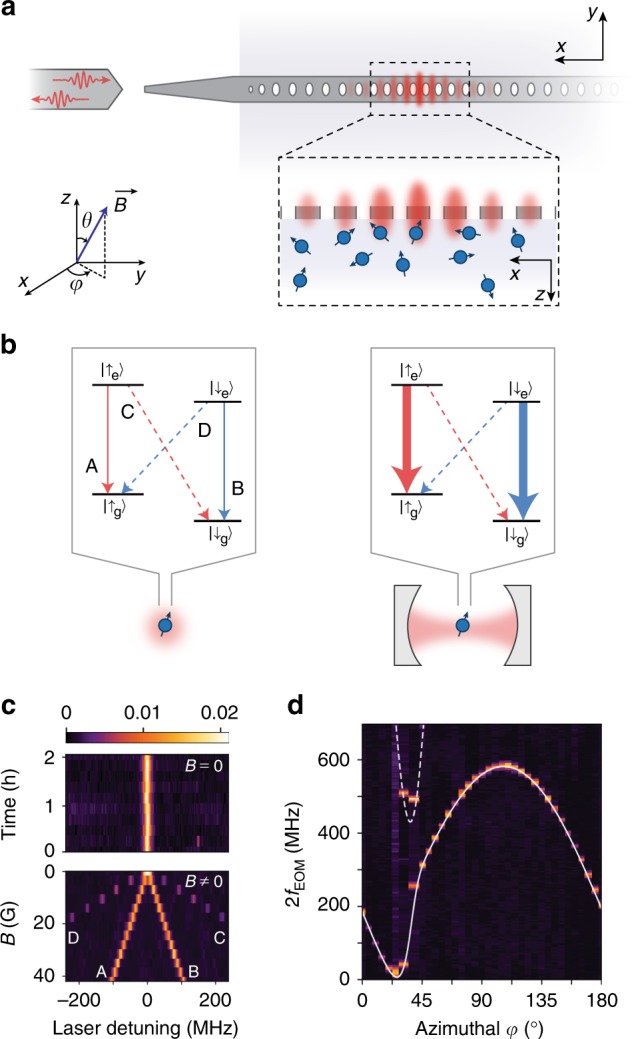
Experimental approach. **a** The experimental device is a silicon photonic crystal cavity on top of an Er^3+^-doped YSO crystal. Light in the cavity evanescently couples to the Er^3+^ ions. (inset) Definition of magnetic field angle (*φ*, *θ*); (*x*, *y*, *z*) refer to the (*D*_1_, *D*_2_, *b*) optical axes of the YSO crystal. **b** Without a cavity, the spin-conserving transitions A, B and spin-non-conserving transitions C, D are comparable in strength. The cavity selectively enhances A,B, resulting in highly cyclic optical transitions. **c** In the absence of a magnetic field, the four transitions are degenerate and give rise to a single, stable optical transition with a full width at half maximum of 6 MHz (centered at *λ* = 1536.48 nm). A magnetic field lifts the degeneracy. The color bar denotes the fluorescence intensity (arbitrary units). **d** The Zeeman splitting is strongly anisotropic, measured here by applying a 112G magnetic field at various angles *φ* (*θ* = 90^∘^) while driving the ion with a phase-modulated laser containing frequencies *f*_0_ ± *f*_EOM_, where *f*_0_ is the transition frequency when *B* = 0. The solid (dashed) line shows the predicted splitting between the A–B (C–D) transitions^29^.

In this work, we demonstrate that tailoring the electromagnetic density of states around an atom with an optical cavity can induce highly cyclic optical transitions in an emitter that is not naturally cyclic. Using a single Er^3+^ ion in Y_2_SiO_5_ (YSO) coupled to a silicon nanophotonic cavity (Fig. 1a), we demonstrate a greater than 100-fold enhancement of the cyclicity: under conditions where the branching ratio of the bare ion results in a spin flip after scattering fewer than ten photons, a cavity-coupled ion can scatter over 1200. This is sufficient to realize single-shot spin readout with a fidelity of 94.6%, and to enable continuous, quantum nondemolition measurement of quantum jumps between the ground state spin sublevels. The improvement in the cyclicity arises from selective Purcell enhancement of the spin-conserving optical decay pathway (Fig. 1b), determined primarily by the alignment of the cavity polarization and the spin quantization axis defined by a magnetic field. A small additional enhancement arises from detuning of the spin-non-conserving transitions from the optical cavity, an effect that was recently used to enhance the cyclicity of a quantum dot in a nanophotonic cavity^27^. This generic technique opens the door to exploiting a much broader range of atomic defects for quantum technology applications, and is a particular advance for individually addressed rare earth ions.

## Results

### Optically addressing single Er^3+^ ions

**Fig. 2:**
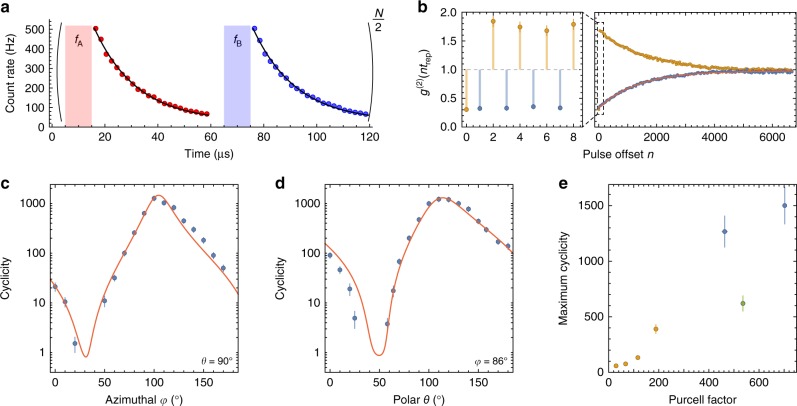
Measuring the cyclicity of the optical transitions. **a** The measurement sequence consists of alternating pulses on the A and B transitions (10 μs) followed by a fluorescence collection window (45 μs), repeating every *t*_rep_ = 60 μs. The fluorescence lifetime is 16.2 ± 0.2 μs, shortened from its free-space value of 11.4 ms by *P* = 703 ± 6 in this device. **b** The intensity autocorrelation *g*^(2)^ is computed from the integrated fluorescence after each pulse. Odd values of *n* (blue points) probe the correlation between pulses driving different transitions and are anti-bunched as a result of the spin staying in the same state over many excitation cycles. *g*^(2)^ decays as ${e}^{-n/{n}_{0}}$ because of optical pumping, giving the cyclicity *C* ≈ *n*_0_/2. In this measurement, *C* = 660 ± 66. *g*^(2)^(0) = 0.3 is consistent with the signal-to-background ratio of 10. **c**, **d** The cyclicity varies dramatically with the angle of the external magnetic field, but is described by a model (red line) based on the independently measured **g** tensors (see text). **e** The cyclicity decreases rapidly with the Purcell factor, demonstrated here by measurements on several ions (shown in different colors) with the detuning varied to change *P*. In all plots, error bars denote estimated  ±1*σ* statistical uncertainty.

Our experimental approach, following ref. ^20^, is based on a YSO crystal doped with a low concentration (<1 ppm) of Er^3+^ ions placed in close proximity to an optical cavity in a silicon photonic crystal waveguide (Fig. 1a). Assembled devices are placed inside a ^3^He cryostat at 0.54 K with a three-axis vector magnet. Light is coupled to the cavities using a lensed optical fiber on a three-axis translation stage. The high quality factor (6 × 10^4^) and small mode volume of the cavity, together with the high radiative efficiency of the Er^3+^ optical transition, enable Purcell enhancement of the Er^3+^ emission rate by a factor of *P* = 700 (Fig. 2a). There are several hundred ions within the mode volume of the cavity, but their optical transitions are inhomogeneously broadened over a several GHz span, such that stable, single ion lines can be clearly isolated (Fig. 1c)^20^.

The ground and excited states of the 1.536 μm optical transition in Er^3+^:YSO are effective spin-1/2 manifolds, which emerge as the lowest energy states of the 16- (14-)fold degenerate ^4^I_15/2_ ground (^4^I_13/2_ excited) free-ion multiplets in the crystal field potential, respectively. In the absence of a magnetic field, the ground and excited states are two-fold degenerate, as required by Kramers’ theorem^28^. This degeneracy is lifted in a small magnetic field, revealing four distinct optical transitions (Fig. 1b, c). Transitions A and B conserve the spin, while C and D flip the spin.

### Improving the cyclicity of the optical transitions

To probe the selection rules of the optical transition, we excite the spin-conserving A and B lines alternately (Fig. 2a). The average fluorescence following the A and B pulses is the same, since the transitions are symmetrically detuned from the cavity and the spin is on average unpolarized from continuous optical pumping by the excitation light. However, the intensity autocorrelation function, *g*^(2)^(*nt*_rep_) (where *n* is the offset in the number of pulses) is anti-bunched for odd-numbered pulse offsets (i.e., A–B correlations) and bunched for even offsets (i.e., A–A or B–B correlations), revealing that only one of the transitions A or B is bright at any given time, depending on the instantaneous spin state (Fig. 2b). Note that the fluorescence after each pulse is integrated before computing the autocorrelation, so *g*^(2)^(*nt*_rep_) is only defined for discrete times. Eventually, the spin relaxes and *g*^(2)^ decays exponentially to 1 after an average of *n*_0_ pulses. Under the assumption (to be verified later) that the observed spin relaxation arises primarily from optical pumping between the spin sublevels, we extract the optical transition cyclicity *C* = *n*_0_*P*_ex_, where *P*_ex_ ≈ 1/2 is the probability to excite the ion in each pulse. This value of *P*_ex_ is assured by using an intense excitation pulse to saturate the ion, and is verified using the independently measured collection efficiency (Supplementary Note 3).

We repeat this measurement with different orientations of the magnetic field, and find that the cyclicity varies by nearly three orders of magnitude (Fig. 2c, d), with a maximum value of 1260 ± 126. This results from the changing orientation of the atomic transition dipole moment with respect to the cavity polarization, with the maximum cyclicity occurring when the spin-conserving transitions A, B are aligned to the cavity and the spin-flip transitions C, D are orthogonal to it. It can be captured by a simple model where the decay rates on each transition are proportional to the projection of an associated dipole moment **d** onto the cavity polarization **ϵ** at the position of the ion (Supplementary Note 2). For the spin conserving transition, ${\Gamma }_{{\rm{AB}}}\propto | \left\langle {\uparrow }_{{\rm{e}}}\right|{\boldsymbol{\epsilon }}\cdot {{\bf{d}}}_{| | }|{\uparrow }_{{\rm{g}}}\rangle {| }^{2}$, while *Γ*_CD_ is defined analogously with **d**_∣∣_ replaced by **d**_⊥_. When the magnetic field is rotated, the spin sublevels mix such that $\left|\uparrow (\varphi ^{\prime} ,\theta ^{\prime} )\right\rangle =\alpha \left|\uparrow (\varphi ,\theta )\right\rangle +\beta \left|\downarrow (\varphi ,\theta )\right\rangle$, with the coefficients *α*, *β* completely specified by the anisotropic **g** tensor describing the Zeeman shifts (Fig. 1d)^29^. Together with the time-reversal symmetry properties of the Kramers’ doublets, this allows the complete angular dependence of *C* = Γ_AB_/Γ_CD_ + 1 to be described by only two parameters: **ϵ** ⋅ **d**_∣∣_ and **ϵ** ⋅ **d**_⊥_ at a single (arbitrary) reference orientation . In this model, the role of the cavity is to restrict the decay to a particular polarization, such that the decay rates are determined by a single matrix element ∣**ϵ** ⋅ **d**∣^2^; in free space, there is no preferred **ϵ**.

Since the dipole matrix elements for Er^3+^:YSO and the cavity field polarization at the position of the atom are not known, we treat **ϵ** ⋅ **d**_∣∣_ and **ϵ** ⋅ **d**_⊥_ as fit parameters. A fit to this model displays excellent agreement with the data, and allows the complete angle dependence of the cyclicity to be extracted from a small number of measurements. While this discussion centers on electric dipole coupling, the Er^3+^ transition we study has comparable electric and magnetic dipole matrix elements^30^, and the predicted magnetic Purcell factor for our structures is similar^20^, depending on the precise position of the ion. We show in the Supplementary Information that the electric and magnetic contributions have the same angular dependence and may be summed into a single term (Supplementary Note 2). We also demonstrate that the detuning of the C, D transitions from the cavity makes an additional, small contribution to the cyclicity at the highest magnetic fields used (Supplementary Note 6).

To quantify the extent to which the cyclicity is enhanced by the cavity, we study a second ion with lower Purcell factor and then lower it further by detuning the cavity. The cyclicity is observed to decrease roughly linearly with *P* (Fig. 2e). Based on the dependence of the cyclicity on the cavity detuning for this ion, we estimate that the cyclicity *C*_0_ of the ion alone is less than 10 (limited by the bare ion branching ratio, which may be affected by decays through intermediate crystal field levels and phonon-assisted excitation to higher excited states; see Supplementary Note 4), such that the enhancement by the cavity is greater than 100. We note that *C*_0_ has not been directly measured for Er^3+^:YSO.

### Single-shot quantum nondemolition measurement

**Fig. 3:**
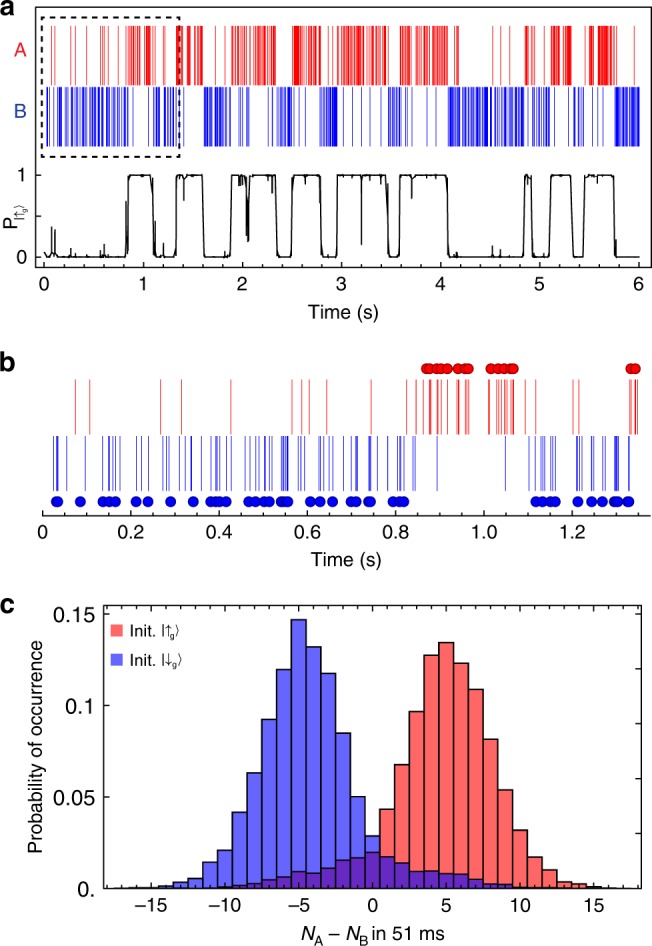
Quantum nondemolition spin measurement. **a** Photons detected during a single run of the experiment using the sequence in Fig. 2a. The black curve shows the probability to be in $|{\uparrow }_{{\rm{g}}}\rangle$ inferred from a Bayesian analysis, revealing quantum jumps between the spin states induced by optical pumping. **b** An adaptive maximum likelihood (ML) algorithm is used to perform successive single-shot spin measurements, with the result of each, independent measurement indicated by a circle [data from dashed box in **a**]. **c** Histogram of photon counts for a fixed integration window of 51 ms following initialization using a ML measurement. The horizontal axis is the difference between the number of A and B photons detected in the measurement window.

Next, we focus on using the cavity-enhanced cyclicity to measure the spin state. Figure 3a shows a time trace of photons recorded in a single run of the experiment, with telegraph-like switching between $|{\downarrow }_{{\rm{g}}}\rangle$ (where transition B is bright) and $|{\uparrow }_{{\rm{g}}}\rangle$ (where transition A is bright) clearly visible. A continuous estimation of the spin state occupation using a Bayesian estimator applied to the full measurement record^31^ shows clearly resolved quantum jumps between these states, demonstrating the quantum nondemolition nature of the measurement. The quantum jumps are driven by optical pumping from the measurement process itself, because of the finite cyclicity.

To demonstrate single-shot measurement of the spin, we use a maximum likelihood (ML) algorithm to estimate the state at time *t* using photon counts from times $t^{\prime} > t$. The measurement duration is adaptive: each measurement terminates when a set fidelity threshold or time limit is reached, and a new, independent measurement is begun^32^. The outcome of each measurement is shown by the circles in Fig. 3b. The average measurement fidelity estimated by the ML algorithm is 94.6%, and 91% of consecutive measurements have the same outcome. The average time to complete a measurement is 20 ms, which corresponds to the average time to detect two photons. The optimum fixed measurement window is 51 ms, resulting in a slower measurement with a lower average fidelity of 91.1% (Fig. 3c).

### Single Er^3+^ spin dynamics

**Fig. 4:**
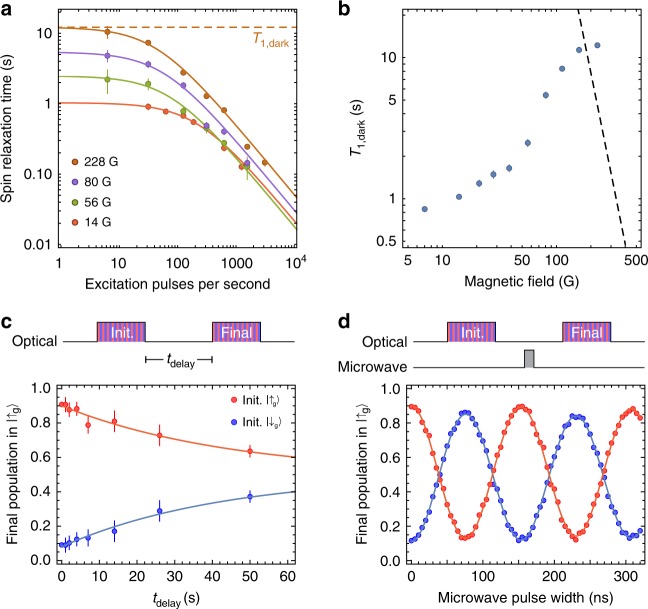
Spin dynamics of a single Er^3+^ ion. **a** Spin relaxation times measured at varying repetition rates of the pulse sequence for several magnetic field amplitudes [(*φ*, *θ*) = (100, 90)°]. At low excitation rates, the spin relaxation time becomes independent of the optical excitation rate, revealing an intrinsic relaxation time *T*_1,dark_. **b** *T*_1,dark_ varies strongly with the amplitude of the magnetic field. The dashed line indicates the expected spin-lattice relaxation rate (Supplementary Note 6)^39^. **c** Using a single-shot projective measurement to initialize the spin, we can directly measure *T*_1,dark_. This experiment is performed on a different ion (ion 3) at 112 G, where *T*_1,dark_ = 45 ± 4 s is roughly five times longer than in the ion used (**a**, **b**). **d** Rabi oscillations can be observed between the two states using a microwave pulse of variable length (*f*_MW_ = 1.76 GHz for *B* = 112 G). **c**, **d** The contrast is consistent with a measurement fidelity of  ~95% for this ion, which enters twice through the initial and final measurements.

Lastly, we apply these spin measurement techniques to investigate the ground state spin dynamics. We infer the intrinsic spin relaxation rate *T*_1,dark_ by reducing the optical excitation rate 1*/t*_rep_ until the total spin lifetime ${T}_{1}=1/({T}_{{\rm{1,dark}}}^{-1}+{T}_{1,{\rm{op}}}^{-1})$, measured via *g*^(2)^, saturates (Fig. 4a). *T*_1,op_ = *Ct*_rep_/*P*_ex_ is the optical pumping time. *T*_1,dark_ increases with increasing magnetic field strength, in a manner that starkly diverges from the expected *B*^−4^ behavior of spin-lattice relaxation (Fig. 4b)^28^. One possible explanation is flip–flop interactions with nearby Er^3+^ ions^33^, which is consistent with the fact that *T*_1,dark_ varies sharply with the magnetic field angle and is different by a factor of 20 between three ions studied (Supplementary Note 6). In this device, the average separation between magnetically equivalent Er^3+^ ions is estimated to be 70 nm, such that the dipole–dipole interaction strength is around 1 kHz; the flip–flop rate is likely much slower because of spectral diffusion from nearby ^89^Y nuclear spins.

In Fig. 4c, we use single-shot spin measurements to directly measure *T*_1,dark_ = 45 ± 4 s in a different ion. This is the longest electronic spin *T*_1_ measured for Er^3+^, to the best of our knowledge^34^. In Fig. 4d, we demonstrate high-visibility Rabi oscillations between the ground state spin sublevels, driven by a microwave magnetic field applied through a coplanar waveguide. We measure ${T}_{2}^{* }=125\pm 5$ ns (in a Ramsey experiment), and *T*_2_ = 3.3 ± 0.2 μs (Hahn echo), consistent with previous measurements of electron spin coherence in solid-state hosts with abundant nuclear spins^17,35^. Longer coherence times to enable storage of quantum states and the observation of coherent dynamics between interacting Er^3+^ ions may be achieved using dynamical decoupling. Ultimately, it will be beneficial to use alternative host crystals with lower nuclear spin content; Er^3+^ incorporation has been demonstrated in several candidates including CaWO_4_, Si, and TiO_2_^36^.

## Discussion

Our results demonstrate that the optical properties of atomic systems are malleable through control of their local environment. Using a photonic nanostructure, we have achieved more than two orders of magnitude improvement in the emission rate and cyclicity of a single Er^3+^ ion, and demonstrated single-shot readout of its spin. Realistic improvements in the quality factor of the optical cavity and photon collection efficiency *η* will enable another 20-fold enhancement in emission rate and spin readout with *F* > 0.99 in 50 μs (*Q* = 10^6^ and *η* = 0.2; see Supplementary Note 5). These results represent a significant step towards realizing quantum networks based on single Er^3+^ ions. This measurement approach may also be extended to address many closely-spaced Er^3+^ spins in the same device by exploiting small differences in their optical transition frequencies, providing a foundation for studying strongly interacting spin systems. Finally, this technique will enable a much broader class of atomic defects to be explored for quantum technologies.

After completion of this work, became aware of a related work demonstrating single-shot spin measurements of single ^171^Yb^3+^ ions coupled to a nanophotonic cavity^37^ using a similar approach.

## Methods

### Devices fabrication

The YSO crystals used in this work were obtained from Scientific Materials, and are doped with trace concentrations of Er^3+^ during growth. Nanophotonic structures are fabricated from silicon-on-insulator wafers using electron beam lithography and inductively-coupled-plasma reactive ion etching (Supplementary Note 1). After undercutting the oxide in concentrated hydrofluoric acid and critical point drying, suspended devices are transferred onto the host crystal using a stamping technique^20^.

### Experimental setup

The assembled devices are installed in a ^3^He cryostat with a base temperature of approximately *T* = 540 mK (spin dynamics are unobservable at *T* = 4 K, presumably because of rapid spin-lattice relaxation in the ground or excited states^38^). For most experiments, light is coupled into the devices using a lensed optical fiber. The measurement of Rabi oscillations in Fig. 4c uses a slightly different device geometry that incorporates a microwave coplanar waveguide approximately 125 μm from the photonic crystal. Microwave pulses are generated using a signal generator modulated by an IQ mixer driven by an arbitrary waveform generator and amplified to 21 W before entering the cryostat. A low duty cycle is used to avoid heating the sample. The optical pulses are derived from a laser stabilized to a ULE reference cavity, and shaped with a sequence of acousto-optic modulators and an electro-optic intensity modulator.

## Supplementary information

Supplementary Information

## Data Availability

The datasets generated during and/or analysed during the current study are available from the corresponding author on reasonable request.
