# Supplementary material for: Optical quantum nondemolition measurement of a single rare earth ion qubit
Source: Nat Commun. 2020 Mar 30;11:1605. doi: 10.1038/s41467-020-15138-7 (PMC7105499; doi:10.1038/s41467-020-15138-7)
Supplement: Supplementary file 1 — Supplementary Information [file 41467_2020_15138_MOESM1_ESM.pdf]

Supplementary Information for "Optical quantum nondemolition measurement of a single rare earth ion qubit"

Mouktik Raha, Songtao Chen, Christopher M. Phenicie, Salim Ourari,
Alan M. Dibos*, Jeff D. Thompson†

Department of Electrical Engineering, Princeton University, Princeton, NJ 08544, USA

February 10, 2020

Supplementary Note 1: Experimental Details

The devices and substrates used in this work are identical to those in Ref. [1], and are described in detail there (Supplementary Fig. 1). The key difference in the present work is that the measurements take place in a ^{3}He cryostat with a base temperature of approximately $T = 540$ mK. At $T = 4$ K, spin dynamics are unobservable, presumably because of rapid spin-lattice relaxation in the ground or excited states [2]. The measurement of Rabi oscillations in Fig. 4c uses a slightly different device geometry that incorporates a microwave coplanar waveguide approximately 125 μm from the photonic crystal. Microwave pulses are generated using a signal generator (SRS SG386) modulated by an IQ mixer driven by an arbitrary waveform generator (Agilent 33622) and amplified to 21 W (MiniCircuits ZHL-30W-252+) before entering the cryostat. A low duty cycle is used to avoid heating the sample.

All data in this paper comes from measurements on 3 different ions (Supplementary Table 1). Figs. 1, 2, 3, Supplementary Figs. 2 and 5b are based on "ion 1", while Fig. 4a,b, Supplementary Figs. 3, 4 and part of Fig. 2e, are based on "ion 2". Fig. 4c,d and part of Fig. 2e are based on "ion 3". Supplementary Fig. 5a contains data from all the above three ions. Ions 1 and 2 are coupled to different photonic crystals on the same YSO substrate, while ion 3 is in a different YSO crystal. Out of the many ions coupled to each cavity, these particular ions were selected for careful study because they are strongly coupled to the cavity (large Purcell factor) and spectrally well-separated from other ions. Importantly, no additional selection was made on the basis of cyclicity or spin readout fidelity. The transition from ion 1 to ion 2 was necessitated by the accidental destruction of the photonic crystal coupled to ion 1 (which was also damaged, lowering Q, after the measurements in Fig. 2a and 3 but before those in Fig. 2c,d), while the transition to ion 3 was motivated by a new device geometry allowing for microwave driving of the spins.

The Er concentration in the YSO substrates used here is estimated to be several hundred ppb. [1], such that the average separation between ions is around 80 nm.

Supplementary Note 2: Theoretical model of cavity-enhanced cyclicity

In this section, we develop a theoretical model describing the cyclicity of the optical transitions measured in Fig. 2 of the main text. Calculating the cavity coupling strength for all four possible transitions A-D is not currently possible because the relevant transition dipole moments of Er^{3+}:YSO have not been measured, and the precise position of the ion in the cavity is not known. As an alternate approach, we demonstrate that these four rates and their dependence on the magnetic field angle can be reduced to only two fit parameters that physically correspond to the decay rates of the AB and CD transitions into the cavity at a single magnetic field orientation. The agreement of this model with the data validates our interpretation of the underlying physics, and is also practically useful for predicting conditions where the cyclicity is maximized from a small number of measurements.

*Present address: Nanoscience and Technology Division, Argonne National Laboratory, Argonne, IL 60439, USA
†jdthompson@princeton.edu

Ion index	Purcell factor	Maximum cyclicity ($C_{\max}$)	Cavity Q factor	Cavity losses (η_{cav})	Total collection efficiency (η)	ML readout fidelity
ion 1 (Fig. 3)	703 ± 6	1500 ± 150	6.6×10^4	0.063	0.028	0.946
ion 1 (Fig. 2)	463 ± 10	1260 ± 126	4.3×10^4	0.045	0.020	-
ion 2	189 ± 5	390 ± 39	7.3×10^4	0.088	0.037	0.83
ion 3	536 ± 5	620 ± 62	4.8×10^4	0.159	0.038	0.968

Supplementary Table 1. **Parameters for the 3 ions studied.** Cavity losses $\eta_{\text{cav}} = \kappa_{\text{wg}}/(\kappa_{\text{wg}} + \kappa_{\text{int}})$, where κ_{wg} and κ_{int} are waveguide and internal loss channels from the cavity, respectively, such that $\kappa_{\text{wg}} + \kappa_{\text{int}} = \kappa$ is the cavity linewidth. η includes η_{cav} as well as the measured fiber-waveguide coupling efficiency (typ. 40-60%), other measured component losses and the detector quantum efficiency (67%).

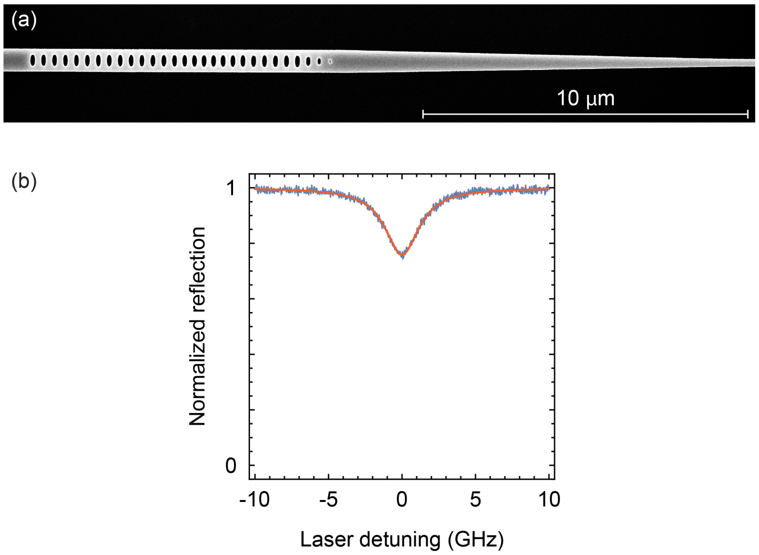

Supplementary Fig. 1. **Device details**. (**a**) Scanning electron microscope image of a representative silicon photonic crystal cavity. The cavity supports a single, non-degenerate high-Q resonance. (**b**) Reflection spectrum of ion 1 cavity (first row in Supplementary Table 1), demonstrating a linewidth $\kappa = 2.85$ GHz.

Supplementary Note 2.1: Atom-cavity Hamiltonian

The Er^{3+} $^4I_{13/2} \to ^4 I_{15/2}$ transition in YSO has roughly equal contributions from electric (ED) and magnetic (MD) dipole transition amplitudes [3]. The Hamiltonian describing this mixed coupling to a field is [4]:

$$H = -\mathbf{d} \cdot \mathbf{E} - \boldsymbol{\mu} \cdot \mathbf{B} \tag{1}$$

Here, $\mathbf{d}$ is the electric dipole operator and $\boldsymbol{\mu}$ is the magnetic dipole operator. Typically, expressions for multipolar coupling include electric quadropole (E2) terms at the same order as MD contributions. However, for Er^{3+}, it has been calculated that the vacuum E2 emission rate is approximately 7 orders of magnitude smaller than the MD rate [3], justifying its exclusion in the present analysis.

We quantize the electric and magnetic fields in the cavity as:

$$\mathbf{E} = \mathbf{E}(\mathbf{r})ae^{-i\omega t} + \mathbf{E}^*(\mathbf{r})a^\dagger e^{i\omega t} \tag{2}$$

$$\mathbf{B} = i\left[\mathbf{B}(\mathbf{r})ae^{-i\omega t} - \mathbf{B}^*(\mathbf{r})a^\dagger e^{i\omega t}\right] \tag{3}$$

The i appearing in the expression for $\mathbf{B}$ reflects the fact that the magnetic field oscillates out of phase with the electric field in a standing wave cavity. For nanophotonic cavities, the TE/TM polarization mode splitting is high enough that we only consider a single polarization mode.

We then introduce four transition dipole operators σ_i, corresponding to the four transitions A-D. Each operator couples to electric and magnetic fields via the dipole moments $\mathbf{d}_i$ and $\boldsymbol{\mu}_i$, respectively. The complete atom-photon interaction Hamiltonian is ($\hbar = 1$):

$$H = -\sum_{i=1}^{4} \left(\mathbf{d}_i\sigma_i + \mathbf{d}_i^*\sigma_i^\dagger\right) \cdot \left(\mathbf{E}(\mathbf{r}_0)ae^{-i\omega t} + \mathbf{E}^*(\mathbf{r}_0)a^\dagger e^{i\omega t}\right) - i\left(\boldsymbol{\mu}_i\sigma_i + \boldsymbol{\mu}_i^*\sigma_i^\dagger\right) \cdot \left(\mathbf{B}(\mathbf{r}_0)ae^{-i\omega t} - \mathbf{B}^*(\mathbf{r}_0)a^\dagger e^{-i\omega t}\right) \tag{4}$$

Making the rotating wave approximation and taking the cavity to be resonant with the atomic transition (neglecting Zeeman splittings), we arrive at:

$$H = -\sum_{i=1}^{4} \left(\mathbf{d}_i \cdot \mathbf{E}^*(\mathbf{r}_0)\sigma_i a^\dagger - i\, \boldsymbol{\mu}_i \cdot \mathbf{B}^*(\mathbf{r}_0)\sigma_i a^\dagger \right) + h.c. \tag{5}$$

In the limit $\kappa \gg (\mathbf{d}\cdot\mathbf{E}, \boldsymbol{\mu}\cdot\mathbf{B})$, the atom-cavity dynamics are simply Purcell-enhanced spontaneous emission into the cavity (the *bad-cavity* limit of cavity QED). The decay rate on the transition i into the cavity is given by:

$$\Gamma_i = \frac{|\mathbf{d}_i \cdot \mathbf{E}^*(\mathbf{r}_0) - i\boldsymbol{\mu}_i \cdot \mathbf{B}^*(\mathbf{r}_0)|^2}{\kappa}, \tag{6}$$

where κ is the cavity linewidth. In previous work, we have shown that the contributions from electric and magnetic coupling to the cavity could be of similar magnitude [1], depending on the position of the ion within in the cavity standing wave.

Supplementary Note 2.2: Reducing parameters using Kramers' theorem

Er^{3+} has an odd number of electrons, so in the absence of a magnetic field, its eigenstates are all even-fold degenerate according to Kramers' theorem. In a low-symmetry environment like the Y site in YSO (C_1 point group), the degeneracy is minimal and all eigenstates are doublets [5]. The application of a magnetic field lifts the degeneracy of the doublets, resulting in the singly degenerate states $\{\downarrow_\mathrm{g}, \uparrow_\mathrm{g}, \downarrow_\mathrm{e}, \uparrow_\mathrm{e}\}$ shown in Fig. 1b.

The states emerging from the same doublet are time-reversal conjugates of each other: $\hat{\Theta}\,|\uparrow_j\rangle = |\downarrow_j\rangle$ and $\hat{\Theta}\,|\downarrow_j\rangle = -\,|\uparrow_j\rangle$, where $\hat{\Theta}$ is the antiunitary time-reversal operator. This has implications for the matrix elements of the electric and magnetic dipole operators, which are even and odd under time reversal ($\Theta^{-1}A\hat{\Theta} = \pm A$), respectively [5]. In particular:

$$\begin{aligned}
\mathbf{d}_1 &= \langle\downarrow_\mathrm{e}|\,\mathbf{d}\,|\downarrow_\mathrm{g}\rangle = \langle\hat{\Theta}\uparrow_\mathrm{e}|\,\mathbf{d}\,|\hat{\Theta}\uparrow_\mathrm{g}\rangle = \langle\uparrow_\mathrm{e}|\,\hat{\Theta}^{-1}\mathbf{d}\hat{\Theta}\,|\uparrow_\mathrm{g}\rangle^* = \langle\uparrow_\mathrm{e}|\,\mathbf{d}\,|\uparrow_\mathrm{g}\rangle^* = \mathbf{d}_4^* & (7)\\
\mathbf{d}_2 &= \langle\downarrow_\mathrm{e}|\,\mathbf{d}\,|\uparrow_\mathrm{g}\rangle = -\,\langle\hat{\Theta}\uparrow_\mathrm{e}|\,\mathbf{d}\,|\hat{\Theta}\downarrow_\mathrm{g}\rangle = -\,\langle\uparrow_\mathrm{e}|\,\hat{\Theta}^{-1}\mathbf{d}\hat{\Theta}\,|\downarrow_\mathrm{g}\rangle^* = -\mathbf{d}_3^* & (8)\\
\boldsymbol{\mu}_1 &= -\boldsymbol{\mu}_4^* & (9)\\
\boldsymbol{\mu}_2 &= \boldsymbol{\mu}_3^* & (10)
\end{aligned}$$

We can now revisit the atom-photon coupling Hamiltonian, Eqn. (5). Writing the atomic operators σ_i in the basis $\{\downarrow_\mathrm{g}, \uparrow_\mathrm{g}, \downarrow_\mathrm{e}, \uparrow_\mathrm{e}\}$:

$$\begin{aligned}
H &= -\begin{pmatrix} 0 & 0 & \mathbf{d}_1 & \mathbf{d}_3 \\ 0 & 0 & \mathbf{d}_2 & \mathbf{d}_4 \\ 0 & 0 & 0 & 0 \\ 0 & 0 & 0 & 0 \end{pmatrix} \cdot \mathbf{E}^*(\mathbf{r_0})a^\dagger + i \begin{pmatrix} 0 & 0 & \boldsymbol{\mu}_1 & \boldsymbol{\mu}_3 \\ 0 & 0 & \boldsymbol{\mu}_2 & \boldsymbol{\mu}_4 \\ 0 & 0 & 0 & 0 \\ 0 & 0 & 0 & 0 \end{pmatrix} \cdot \mathbf{B}^*(\mathbf{r_0})a^\dagger + h.c. & (11)\\
&= -\begin{pmatrix} 0 & 0 & \mathbf{d}_{||} & \mathbf{d}_\perp \\ 0 & 0 & -\mathbf{d}_\perp^* & \mathbf{d}_{||}^* \\ 0 & 0 & 0 & 0 \\ 0 & 0 & 0 & 0 \end{pmatrix} \cdot \mathbf{E}^*(\mathbf{r_0})a^\dagger + i \begin{pmatrix} 0 & 0 & \boldsymbol{\mu}_{||} & \boldsymbol{\mu}_\perp \\ 0 & 0 & \boldsymbol{\mu}_\perp^* & -\boldsymbol{\mu}_{||}^* \\ 0 & 0 & 0 & 0 \\ 0 & 0 & 0 & 0 \end{pmatrix} \cdot \mathbf{B}^*(\mathbf{r_0})a^\dagger + h.c. & (12)\\
&= -\begin{pmatrix} 0 & 0 & g_{||}^e - ig_{||}^m & g_\perp^e - ig_\perp^m \\ 0 & 0 & -g_\perp^{e\,*} - ig_\perp^{m\,*} & g_{||}^{e\,*} + ig_{||}^{m\,*} \\ 0 & 0 & 0 & 0 \\ 0 & 0 & 0 & 0 \end{pmatrix} a^\dagger + h.c. & (13)\\
&= -\begin{pmatrix} 0 & 0 & g_{||} & g_\perp \\ 0 & 0 & -g_\perp^* & g_{||}^* \\ 0 & 0 & 0 & 0 \\ 0 & 0 & 0 & 0 \end{pmatrix} a^\dagger + h.c. & (14)
\end{aligned}$$

In going from the first to the second line, we have made use of Eqns. (7) - (10). In going from the second to the third line, we have taken the cavity fields to be real-valued, which is an excellent approximation as they are highly linearly polarized.

The final expression, Eqn. (14), has the same form for the electric and magnetic dipole moments despite the difference in their transformation properties in Eqns. (7) - (10). Mathematically, this arises from the factor of i in front of the magnetic field term in Eqn. (5). Physically, this means that interference of the electric and magnetic dipole decay channels into the cavity does not introduce any chirality (*i.e.*, preference for decays from $|\uparrow_{\rm e}\rangle$ relative to $|\downarrow_{\rm e}\rangle$) into the atom-cavity system, which is intuitive for a standing-wave optical cavity.

Supplementary Note 2.3: Angle dependence of the cyclicity

We now turn to computing the cyclicity and its dependence on the orientation of the external magnetic field defining the spin quantization axis. The branching ratio between spin-non-conserving and spin-conserving decays through the cavity is $R = \Gamma_\perp/(\Gamma_{||} + \Gamma_\perp) = |g_\perp|^2/(|g_{||}|^2 + |g_\perp|^2)$, and the cyclicity is $C = 1/R$. The spin eigenstates are given by the effective spin Hamiltonian:

$$H_Z = \mu_{\rm B}\mathbf{B}\cdot\mathbf{g}\cdot\mathbf{S}, \tag{15}$$

where $\mathbf{B}$ is the applied magnetic field, $\mathbf{S} = \{\sigma_x, \sigma_y, \sigma_z\}$ is a vector of Pauli matrices, $\mu_{\rm B}$ is the Bohr magneton and $\mathbf{g}$ is a symmetric, real 3x3 matrix. For Er^{3+}:YSO, $\mathbf{g}$ is highly anisotropic in both the ground and excited electronic states, with principal components $(14.65, 1.80, 0.56)$ for the ground state and $(12.97, 0.85, 0.25)$ for the excited state [6]. Note that the orientation of the eigenvectors of $\mathbf{g}$ is different from the crystal axes and also slightly different between the ground and excited states. A consequence of the anisotropy of $\mathbf{g}$ is that the spin eigenvectors are not generally parallel to the applied magnetic field.

The eigenvectors at one magnetic field orientation (φ', θ') can be expressed in terms of those at another orientation as: $|\uparrow_{\rm g}(\varphi',\theta')\rangle = \alpha\,|\uparrow_{\rm g}(\varphi,\theta)\rangle + \beta\,|\downarrow_{\rm g}(\varphi,\theta)\rangle$. Denoting the matrix of atom-cavity coupling matrix elements in Eqn. (14) as $M(\varphi,\theta)$, we can transform it to another basis according to:

$$g_{||}(\varphi',\theta') = \langle\downarrow_{\rm g}(\varphi',\theta')|\,M(\varphi,\theta)\,|\downarrow_{\rm e}(\varphi',\theta')\rangle \tag{16}$$

$$g_{\perp}(\varphi',\theta') = \langle\downarrow_{\rm g}(\varphi',\theta')|\,M(\varphi,\theta)\,|\uparrow_{\rm e}(\varphi',\theta')\rangle \tag{17}$$

and evaluate the cyclicity as before.

We note that a similar model was applied to the orientation dependence of the *absorption* of linearly polarized light by Nd^{3+}:YVO_4 in Ref. [7]. The applicability of this model to *emission* in our work stems from the restriction of the emission to a single polarization by the high Purcell factor coupling to a single-mode, non-degenerate cavity.

This model is used to fit the data in Fig. 2 of the main text. The model is fit using $M(100^\circ, 90^\circ)$ as the fit parameters, and the result is $g_{||} = e^{-i1.15}$, $g_\perp = 0.024 \times e^{-i1.476}$. Since the cyclicity only depends on the ratio of these quantities, we constrain $|g_{||}| = 1$.

Supplementary Note 2.4: Correction for free-space decay

As the Purcell factor is finite, free-space emission can influence the cyclicity when it is very large. We incorporate this by adding free space decays to the branching ratio expression, such that the cyclicity becomes C_0 when the cavity coupling vanishes:

$$C = \frac{\Gamma_{\rm CD} + \Gamma_{\rm AB}}{\Gamma_{\rm CD}} = 1 + \frac{\Gamma^0_{\rm AB} + |g_{||}|^2/\kappa}{\Gamma^0_{\rm CD} + |g_\perp|^2/\kappa} = 1 + \frac{1 - 1/C_0 + P_{||}}{1/C_0 + P_\perp} \tag{18}$$

In this expression, Γ^0_i denotes the decay rate on transition i in the absence of a cavity, $C_0 = 1 + \Gamma^0_{\rm AB}/\Gamma^0_{\rm CD}$ is the cyclicity in the absence of a cavity, and $P_{||/\perp} = g^2_{||/\perp}/(\kappa\Gamma^0)$, where Γ^0 is the total decay rate out of the excited state in the absence of the cavity.

For the ions studied here with P of order several hundred, the inclusion of this correction does not meaningfully improve the fit. We believe this occurs because the fit function can artificially increase $g_\perp$ by a small amount at the orientation of maximum cyclicity to account for the free space decay without significantly impacting the cyclicity at other angles. However, measuring the same ion with different Purcell factors (realized by changing the cavity detuning) makes the free-space decay evident and allows C_0 to be estimated (Supplementary Note 4).

Supplementary Note 2.5: Highest achievable cyclicity

For an atom with Purcell factor P and bare cyclicity C_0, the highest possible cyclicity is $C = PC_0$, achieved when $g_\perp = 0$. This can always be realized by choosing the cavity polarization to be perpendicular to $\mathbf{d}_\perp$, for some spin quantization axis (although in the special case that $\mathbf{d}_{||}||\mathbf{d}_\perp$, P will also vanish). In contrast, if the cavity polarization is fixed, it may be possible to tune the quantization axis angle to achieve $g_\perp = 0$. We consider three cases:

1. If the **g** tensors in the ground and excited state are isotropic (or equal), it is always possible to choose an orientation of **B** where $g_\perp = 0$. This can be proved from the normality of the matrix m formed by the upper-right 2x2 block of M.

2. If the **g** tensors for the ground and excited states are not simultaneously diagonalizable (having different axes), then it is not possible to achieve $g_\perp = 0$ in general. This is proved by the specific counterexample of Er^{3+}:YSO, where we observe (in a numeric model) that $g_\perp$ can be made small but never quite zero for certain values of M.

3. If the **g** tensors for the ground and excited states are parallel but have different (non-zero) magnetic moments, it appears (numerically) to always be possible to make $g_\perp = 0$. We have not proven this mathematically.

Er^{3+}:YSO falls into the second case because of the C_1 site symmetry of the Er site. However, the principal axes of the ground and excited state **g** tensors are only rotated by about 15° from each other [6], which may explain the success in achieving high cyclicity anyway. Many other host crystals for REIs and other defects have higher site symmetry, which forces the alignment of the g tensor axes to the crystal, and are therefore covered by the third case. Therefore, we expect that this technique is fairly general.

Supplementary Note 2.6: Correction of cyclicity estimate at small n_0 values

In Fig. 2b and thereafter, the intensity autocorrelation $g^{(2)}$ has been fitted to an exponential function and the decay constant n_0 is utilized to extract $C = P_{\text{ex}} n_0$. This expression for C is only valid when $n_0 \gg 1$ because of the discrete time steps in the measurement. We estimate the value of C more accurately by using the following expression:

$$C = \frac{P_{\text{ex}}}{\left(1 - e^{-1/n_0}\right)}, \tag{19}$$

which reduces to the previous expression for C at large n_0 values.

Supplementary Note 2.7: Extracting n_0 from $g^{(2)}$ at low optical pumping rates

The spin relaxation time is extracted from fits to $g^{(2)}$ measurements as described in Fig. 2b. When these time constants are longer than 1 s, the even- and odd-offset $g^{(2)}$ time constants begin to differ. We believe this results from spectral diffusion of the optical transitions. Under the assumption that this is uncorrelated with the spin dynamics and acts identically on the A and B transitions, we find that the spin time constant can be isolated by fitting an exponential to the *difference* of the even- and odd-offset traces.

Supplementary Note 3: Calibration of P_{ex}

For the measurements in Fig. 2 of the main text, we use saturating optical pulses to ensure $P_{\text{ex}} \approx 0.5$ regardless of the magnetic field orientation. The approximate value of P_{ex} is confirmed by counting the emitted photons and comparing to the independently measured collection and detection efficiency (Supplementary Fig. 2). We note that the finite duration of the excitation pulse may allow for $P_{\text{ex}} > 0.5$ if a photon is emitted during the pulse and the ion is re-excited, and have observed increased values of C at certain angles when using optical π pulses instead. In that sense, the values of C that we quote should be interpreted as lower bounds.

Supplementary Note 4: Estimate of intrinsic cyclicity of Er^{3+}:YSO transition

A central claim of our work is that the cyclicity of the ion is enhanced by the optical cavity. Since the cyclicity C_0 of the spin transitions in Er^{3+}:YSO without a cavity has not been previously measured, there is no direct basis for comparison. In ensemble experiments, C_0 has been estimated to be around 10 using measurements of the optical pumping rate and a number of simplifying assumptions [8]. We have attempted to measure C_0 using a single ion by detuning the cavity as much as possible, which increases the fraction of decays into free space and yields a weighted average of the cavity-enhanced C and C_0 [Eq. 18].

In Supplementary Fig. 3c, we show several measurements of the same ion with increasing cavity detuning to decrease the total Purcell factor. The maximum cyclicity is strongly reduced: at the highest detuning, we observe a

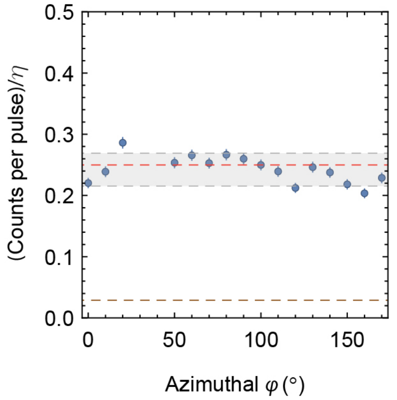

Supplementary Fig. 2. $P_{\mathbf{ex}}$ **calibration**. Average number of collected photons following each excitation pulse during the measurement in Fig. 2c. The photon numbers are scaled by the independently measured collection and detection efficiency $\eta = 0.020$. Since the spin is on average unpolarized and only resonant with the laser half of the time, $P_{\text{ex}} = 1/2$ (as defined here) corresponds to 1/4 photon per pulse. In all plots, error bars denote estimated $\pm 1\sigma$ statistical uncertainty.

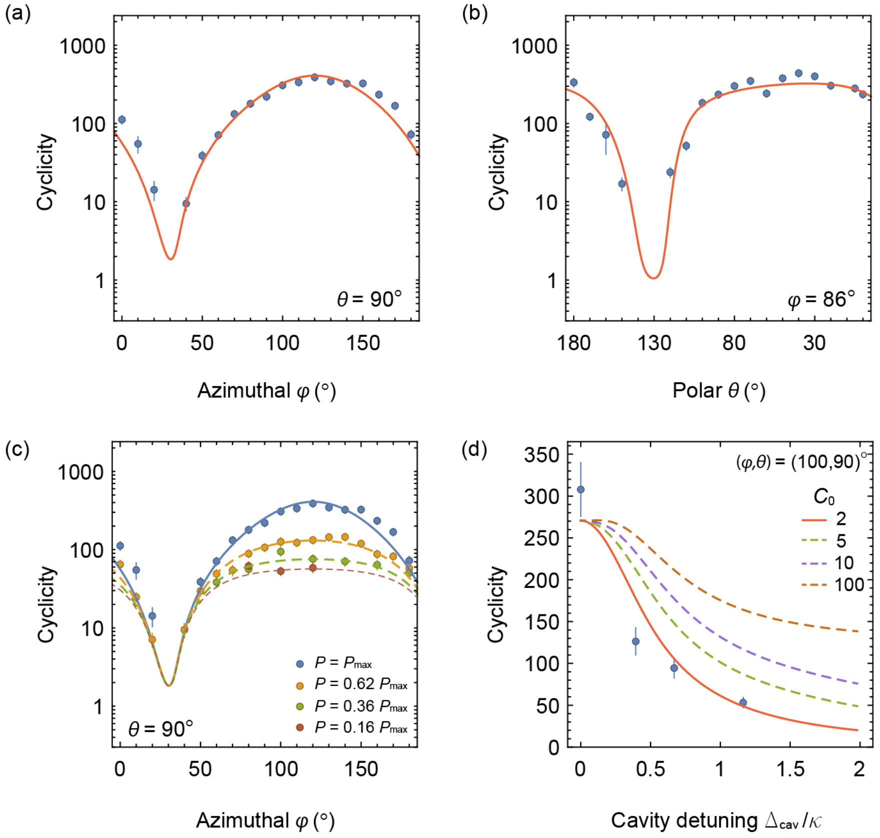

Supplementary Fig. 3. **Extracting the bare ion cyclicity,** C_0. (**a**,**b**) Cyclicity measurements on ion 2 (situated in a different cavity) show a similar orientation dependence to ion 1. Each of the two crystallographically inequivalent Y sites in YSO has two possible orientations related by a C_2 rotation about the b axis. Ion 2 is in the opposite site from ion 1, so we have inverted the θ axis to make the apparent dependence the same. (**c**) Measurement of cyclicity for the same ion 2 at several different Purcell factors, achieved by detuning the cavity from the atomic transition by an amount Δ_{cav}. The data is fit to Eq. 18. Here, $B = 112$ G and $\theta = 90°$. (**d**) Cyclicity at $(\varphi, \theta) = (100, 90)°$ vs. cavity detuning. The model is Eq. 18, where $P_{||}$ and $P_{\perp}$ acquire a detuning dependence $P(\Delta_i) = \frac{P_{\max}}{1+(2\Delta_i/\kappa)^2}$ based on the known Zeeman splittings of the four transitions. Since the decay rates out of the two excited states are no longer equal when the cavity is detuned (*i.e.*, $\Gamma_{\text{A}} \neq \Gamma_{\text{B}}$), Eq. 18 is averaged over the two excited states. A fit yields $C_0 = 2 \pm 3$.

cyclicity of 53 ± 5, which sets an upper bound on C_0. In Supplementary Fig. 3d, we plot the cyclicity vs. detuning together with a theoretical model for several values of C_0. From this model, we conclude that C_0 is less than 10, and likely closer to 2. Direct measurements at higher detunings are not possible because the count rate falls dramatically.

Supplementary Note 5: Single-shot measurement fidelity

Two factors contribute to the ML fidelity: the statistical error set by the signal-to-background ratio during the collection period (SNR), and the probability to decay to the wrong state before enough photons are collected, $m/(\eta C)$, where m is the number of photons needed to reach the target fidelity F_{T}. m is related to the SNR as $F_{\mathrm{T}} = SNR^m/(SNR^m + 1)$. In our experiment, SNR is typically high enough (14 for ion 1, limited by a timing error, and around 20 for ions 2 and 3, limited by dark counts) that by the time a single photon is detected, the error from the finite cyclicity is larger than the statistical error. In this regime, a nearly-optimal strategy is to infer that the spin state is $|\uparrow_{\mathrm{g}}\rangle$ whenever an "A" photon is detected, and $|\downarrow_{\mathrm{g}}\rangle$ whenever an "B" photon is detected. The average measurement fidelity is $1 - 1/(\eta C)$, and the average measurement duration is $t_{\mathrm{meas}} = 1/(P_{\mathrm{ex}}\eta C)$.

Based on this model, we can project how improved devices might lead to improved measurements. Assuming the same demonstrated cyclicity (1500), fiber-waveguide coupling and photon detection efficiency, but improving the cavity to realize $Q_{\mathrm{int}} = 10^6$ with critical coupling ($\kappa_{\mathrm{wg}} = \kappa_{\mathrm{int}}$) and using optical π pulses to increase P_{ex} to 1, it should be possible to realize an average measurement fidelity of 0.996 in average time of 50 μs. This assumes that SNR remains dark-count limited as the Purcell factor is increased.

Supplementary Note 6: Intrinsic spin relaxation

The spin relaxation rates in Fig. 4a are fit to a model of the form $T_1^{-1} = T_{1,\mathrm{dark}}^{-1} + P_{\mathrm{ex}}/(t_{\mathrm{rep}}C)$, where the repetition rate t_{rep} is varied to change the optical pumping strength. The intrinsic relaxation rate $T_{1,\mathrm{dark}}$ varies strongly with the magnetic field amplitude, and the cyclicity C also has a weak dependence. The latter is explained by the larger Zeeman shift of the spin-non-conserving transitions CD ($\sim$ 13.1 MHz/Gauss) compared to the AB transitions ($\sim$ 2.5 MHz/Gauss) shifting the former out of resonance with the cavity more quickly (Supplementary Fig. 4). From this data, we can also affirm that the selective Purcell enhancement of the spin-conserving transition does not primarily arise from detuning the CD transitions, as in Ref. [9], although this effect does provide an additional factor of 2-3 at the highest magnetic fields.

The magnetic field dependence of $T_{1,\mathrm{dark}}$ disagrees markedly with predictions based on the measured coefficients for the Raman, Orbach and direct processes for Er^{3+}:YSO [10]. At $T = 0.5$ K, these are dominated by the direct process, which should have a magnetic field dependence $T_1 \propto B^{-4}$, which arises from the combination of the frequency-dependence of the phonon density of states and the magnetic field-dependence of the spin-phonon coupling [5]. The measured values display a $B^{1/2}$ dependence at low fields, transitioning to a more rapid increase around $B = 50$ Gauss before saturating at 200 Gauss. We note that the point where $T_{1,\mathrm{dark}}$ saturates is roughly consistent with the onset of the direct process, and that the disagreement could be attributed to anisotropy in

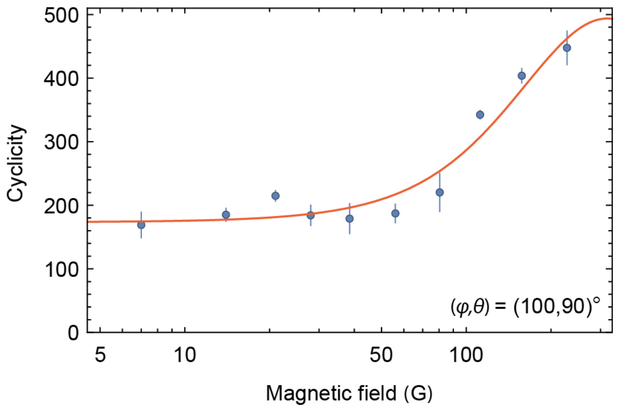

Supplementary Fig. 4. **Magnetic field amplitude dependence of cyclicity**. Based on the data for Ion 2 in Fig. 4a,b of the main text, we extract the cyclicity of the optical transitions at a fixed magnetic field orientation for varying field amplitudes. The data is fit to the model in Eq. 18, incorporating the detuning-dependence of the Purcell enhancement as described in the caption to Fig. 3. C_0 fits here to 5 ± 1.

the direct process rate. Anomalous magnetic field dependence in the low-field relaxation of rare earth ions has been previously observed in electron paramagnetic resonance [11] without a definitive explanation. Concentration-dependent spin relaxation has been observed in spectral hole burning experiments [12] and attributed to flip-flop interactions between nearby Er ions. This is a likely explanation for our observations, which is bolstered by the difference in $T_{1,\mathrm{dark}}$ for different ions [which may arise from the stochastic arrangement of ions in this low-density sample ([Er] $\approx$ 300 ppb)] and a strong ansiotropy in $T_{1,\mathrm{dark}}$ (Supplementary Fig. 5).

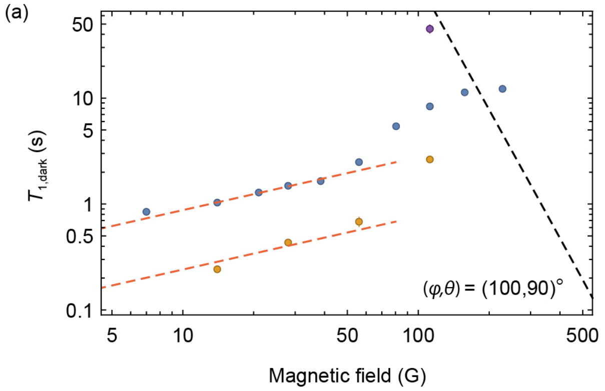

Supplementary Fig. 5. **Additional measurements of $T_{1,\mathrm{dark}}$.** (**a**) Intrinsic spin relaxation time ($T_{1,\mathrm{dark}}$) of ion 1 (yellow) measured under the same conditions as ion 2 (blue points, reproduced from Fig. 4b). $T_{1,\mathrm{dark}}$ for ion 3 (purple point, reproduced from Fig. 4c), measured for a field oriented along $(\varphi, \theta) = (130, 90)^\circ$, is also included here. The empirical $B^{1/2}$ scaling observed at low fields is indicated by the red, dashed line. The relaxation rate for ion 1 follows a similar trend to ion 2 but is 4 times faster. The black, dashed line is the model from Ref. [10]. (**b**) $T_{1,\mathrm{dark}}$ (green points) measured at a few field orientations ($B = 28$ G) reveal the anisotropic nature of $T_{1,\mathrm{dark}}$. The angle dependence qualitatively agrees with flip-flop limited Er^{3+}:YSO lifetimes measured in Ref. [12]. The brown points denote the optical pumping time constants under these conditions, showing that the cyclicity measurements are generally not limited by $T_{1,\mathrm{dark}}$.

Supplementary References

1. Dibos, A. M., Raha, M., Phenicie, C. M. & Thompson, J. D. Atomic Source of Single Photons in the Telecom Band. *Phys. Rev. Lett.* **120,** 243601 (2018).
2. Welinski, S. *et al.* Electron Spin Coherence in Optically Excited States of Rare-Earth Ions for Microwave to Optical Quantum Transducers. *Phys. Rev. Lett.* **122,** 247401 (2019).
3. Dodson, C. M. & Zia, R. Magnetic dipole and electric quadrupole transitions in the trivalent lanthanide series: Calculated emission rates and oscillator strengths. *Phys. Rev. B* **86,** 125102 (2012).
4. Barron, L. D. *Molecular light scattering and optical activity* (Cambridge University Press, Cambridge, 2004).
5. Abragam, A. & Bleaney, B. *Electron paramagnetic resonance of transition ions* (Dover, New York, 1986).
6. Sun, Y., Böttger, T., Thiel, C. W. & Cone, R. L. Magnetic g tensors for the $^4I_{15/2}$ and $^4I_{13/2}$ states of Er^{3+}:Y_2SiO_5. *Phys. Rev. B* **77,** 085124 (2008).
7. Afzelius, M. *et al.* Efficient optical pumping of Zeeman spin levels in Nd^{3+}:YVO_4. *J. Lumin.* **130,** 1566–1571 (2010).
8. Hastings-Simon, S. R. *et al.* Zeeman-level lifetimes in Er^{3+}:Y_2SiO_5. *Phys. Rev. B* **78,** 085410 (2008).
9. Sun, S., Kim, H., Solomon, G. S. & Waks, E. Cavity-Enhanced Optical Readout of a Single Solid-State Spin. *Phys. Rev. Appl.* **9,** 054013 (2018).
10. Kurkin, I. N. & Chernov, K. P. EPR and spin-lattice relaxation of rare-earth activated centres in Y_2SiO_5 single crystals. *Phys. B+C* **101,** 233–238 (1980).
11. Sabisky, E. S. & Anderson, C. H. Spin-Lattice Relaxation of Tm^{2+}in CaF_2, SrF_2, and BaF_2. *Phys. Rev. B* **1,** 2028–2040 (1970).
12. Car, B., Veissier, L., Louchet-Chauvet, A., Gouët, J.-L. L. & Chanelière, T. Optical study of the anisotropic erbium spin flip-flop dynamics. *Phys. Rev. B* **100,** 165107 (2019).
